# Anatomical reduction and precise internal fixation of intra-articular fractures of the distal radius with virtual X-ray and 3D printing

**DOI:** 10.1007/s13246-019-00795-w

**Published:** 2019-10-22

**Authors:** Jing Xu, Guodong Zhang, Zaopeng He, Shizhen Zhong, Yongshao Chen, Chunrong Wei, Yudong Zheng, Haibin Lin, Wei Li, Wenhua Huang

**Affiliations:** 1 National Key Discipline of Human Anatomy, School of Basic Medical Sciences, Southern Medical University, No. 1838 Guangzhou Road North, Guangzhou, 510515 Guangdong China; 2 Hand and Foot Surgery & Plastic Surgery, Affiliated Shunde Hospital of Guangzhou Medical University, No. A163, Middle Road, Lecong Avenue, Shunde District, Foshan, 528315 Guangdong China; 3 Department of Orthopedics, Affiliated Hospital of Putian University, No. 999 East Shenzhen Road, Licheng District, Putian City, 510080 Fujian China; 4 Affiliated Shunde Hospital of Guangzhou Medical University, No. A163, Middle Road, Lecong Avenue, Shunde District, Foshan, 528315 Guangdong China

**Keywords:** Distal radial fracture (DRF), Intra-articular distal radial fracture (IDRF), Anatomical reduction, Internal fixation, Virtual X-ray, 3D printing

## Abstract

To evaluate and precisely internal fix intra-articular distal radial fracture (IDRF) using the virtual X-ray and three-dimensional (3D) printing technologies. Twenty-one patients with IDRF were recruited, and the data from digital design group (DDG) and real surgery group (RSG) were collected and analyzed. In DDG, the data from thin-slice computed tomography scan, virtual X-ray measurement parameters, including volar tilt, palmar tilt, radius length (D1), ulnar variation (D2), locking plate position parameter (D3) and distance between key nail and joint surface (D4) were collected. The bone was virtually fixed with the locking plate, and the final model of radius with the screw was obtained by 3D printing. In RSG, the locking plate was precisely pre-bended and used in surgery. During the surgery, the key K-wire was accurately placed and the locking plate was adjusted with the aid of the U-shaped navigation arm. The C-arm was used to observe the positions of key K-wires and the locking plate, and the same above-mentioned parameters were measured intra- and post-operatively. The data from RSG and DDG were compared statistically by t test. This approach proved to be successful in all 21 patients, and none of the screws pierced through the wrist joint surface. All the measured parameters, including the volar tilt, palmar tilt, D1–4, in RSG were not significantly different from preoperative DDG data. Virtual X-ray measurement of anatomical reduction parameters and 3D printing can help the anatomical reduction and precise internal fixation by providing quantitative references, preoperatively, intraoperatively and postoperatively.

## Introduction

The anatomical structure adjacent to the distal radius is very complex, which is directly related to the hand function [1, 2]. Distal radial fracture (DRF) is a common fracture in the wrist, accounting for about 17% of emergency fractures. Intra-articular DRF (IDRF), a severe form of DRF, accounts for about 25% of total DRF [3]. Currently, it is still very challenging to completely recover from the IDRF, because it is necessary to achieve not only an anatomical reduction [4, 5], i.e., complete reconstruction of radius and the articular surface [6], but also a strong and precise internal fixation to facilitate early rehabilitation [7]; otherwise complications such as traumatic arthritis will often occur, which will seriously affect the function of the hands and wrists [8, 9].

As mentioned above, for orthopedic surgeons, the most challenging issues are still the anatomical reduction and precise internal fixation, even though there are also some controversies on the diagnosis, imaging assessment, treatment and rehabilitation for IDRF [10]. To overcome this challenge, different groups have proposed and tested different approaches. Some investigators focused on improving the surgical procedure or entry route itself, for example, Gao et al. used the extended flexor carpi radialis approach to avoid the complications of double entry (i.e., combined volar and dorsal entry) surgical approach [11]. Safdari et al. compared the open reduction and internal fixation, and found that volar plate fixation, is a more preferred surgical procedure than the external fixator for the treatment of IDRF [12]. However, Shukla et al. found that external fixation showed superiority over volar locked plating after 1 year of surgery [13]. Hardman et al., on the other hand, found that fixed angle intramedullary fixation devices facilitate reduction, with radiological and clinical parameters at least equivalent to volar plate devices [14]. In addition, Khan et al. found that use of locking compression plate for IDRF showed good results [15]. Along the same line, Calbiyik et al. compared the volar locking plate versus intramedullary nailing for fixation and found that both methods have some advantages and shortcomings. For example, intramedullary nailing provides better restoration of volar tilt and wrist flexion, while volar plate fixation provides better restoration of radio-ulnar variance and wrist supination [16]. However, all these studies apparently suggested that none of the currently available modifications of surgical procedure would completely solve the potential problems.

Therefore, other investigators tried to improve the efficiency of precise anatomical reduction and reduce the tissue damage by taking advantage of available cutting-edge tools developed for micro-invasive surgeries, such as fluoroscopically-assisted and arthroscopically-assisted approaches. Unfortunately, in this area, controversies also exist. For example, many investigators agree that arthroscopic reduction of intra-articular fragments is superior to reduction under fluoroscopy. However, Varitimidis et al. argued that the combination of arthroscopy with the fluoroscopically-assisted treatment of IDRF improves the clinical outcome [17]. Nevertheless, Thiart et al. found that IDRF can be successfully treated with fluoroscopically-assisted fragment specific fixation only, and arthroscopy didn’t improve much on top of this treatment regime [18].

To further improve the procedure, some investigators blended above-mentioned surgical procedure modifications with that of micro-invasive technique, developing some new methods, and one method called plate presetting arthroscopic reduction technique (PART). The advantage of PART is that it allows detection of intra-articular movement of fracture fragments, soft tissue lesions such as scapholunate ligament tears and injuries of the triangular fibrocartilage complex (TFCC), screw protrusion, and associated soft tissue injuries [19, 20]. However, all available options still have noticeable problems and therefore none of them have been commonly accepted gold standard.

In this study, we explored a new approach by designing a digital surgical method based on virtual engineer, including thin-layer computed tomography (CT) scan data, virtual three-dimensional (3D) reconstruction and 3D printing, which could simultaneously realize the quantitative evaluation, accurate anatomical reduction and internal fixation of IDRF. Therefore, we argued that this computer-aided design and computer-aided manufacturing (i.e., 3D printing), could be tremendously helpful for this challenging surgical procedure, including in the preoperative preparation and planning, intraoperative guide and postoperative quantitative assessment.

In this approach, several virtual engineering tools are essential, including the virtual 3D reconstruction, virtual X-ray, CT scan and “3D printing”. The virtual 3D reconstruction is the creation of 3D models usually from a set of 2D images. The virtual X-ray is the reverse process of 3D reconstruction, i.e., the virtual X-ray essentially obtaining 2D images from 3D scenes. CT, a computer-assisted imaging process, can produce cross-sectional images (virtual “slices”) of specific areas of a scanned object, using many X-ray measurements taken from different angles. “3D printing” normally referred to an additive manufacturing technique that could produce 3D objects layer by layer through depositing a binder material with inkjet printer head.

Those virtual engineering tools are not completely new. It has been widely used for diagnosis or treatment planning in a variety of different clinical contexts already. For example, preoperative computer simulation has been used in treating complex pelvic and acetabular fractures with reasonable success rate [21]. However, virtual X-ray and 3D printing technology have not been systematically tested in helping anatomical reduction and precision internal fixation of IDRF; therefore, we specifically tested this approach in this study.

## Materials and methods

### Patients’ data

The study was approved by the Institutional Review Board of Affiliated Shunde Hospital of Guangzhou Medical University. Twenty-one cases of adult IDRF were recruited from our hospital (Affiliated Shunde Hospital of Guangzhou Medical University) from February 1, 2017 to May 1, 2018. The general clinical information was summarized in Table  1.

**Table 1:** Summary of the general clinical information of recruited patients

	Males	Females
Total (*n*)	19	2
Age (years)	38.3 ± 10.83	40.1 ± 11.2
Fracture side (*n*)		
Left	11	1
Right	8	1
AO/OTA classification (*n*)		
C1	7	0
C2	11	2
C3	1	0

The indications for surgery were based on the American Association of Orthopaedic Surgeons (AAOS) standard [22].

#### Inclusion criteria

(1) The comminuted fracture of the distal radius confirmed by clinical imaging. (2) The shortening of distal radius > 3 mm after manual reduction (3) the angle of distal radius toward the dorsal side > 10°, or (4) surgical treatment of intra-articular fractures with displaced or stepped irregularities > 2 mm.

#### The exclusion criteria

Patients with mental disorders, severe cardiovascular and cerebrovascular diseases who cannot tolerate surgery.

### Experimental materials and methods

#### Software

(1) Mimics 14.0 and 19.0 (Materialise, Belgium), and (2) SolidWorks 2011 (Dassault Systemes S.A, France).

#### Hardware

(1) 3D printer: Zortrax M200 3D printer (Zortrax, Poland). (2) CT: Philips/Ingenuity CT (Philips, Netherlands). The conditions for thin-wall CT scans were set to: voltage 100 kV, current 175 mA, pitch 0.5 mm, layer thickness 0.625 mm, and the Dicom format image was output to Mimics 19.0 through the PACS system.

#### Experimental design

For each patient, two groups of data, including the volar tilt, palmar tilt, D1–4, were obtained and analyzed; one was obtained mainly through thin-wall CT scans during virtual reduction (termed digital design group, DDG), and the other group of data of the same parameters was obtained during real surgery (termed real surgery group, RSG).

#### Sequential virtual fracture reduction

**Fig. 1:**
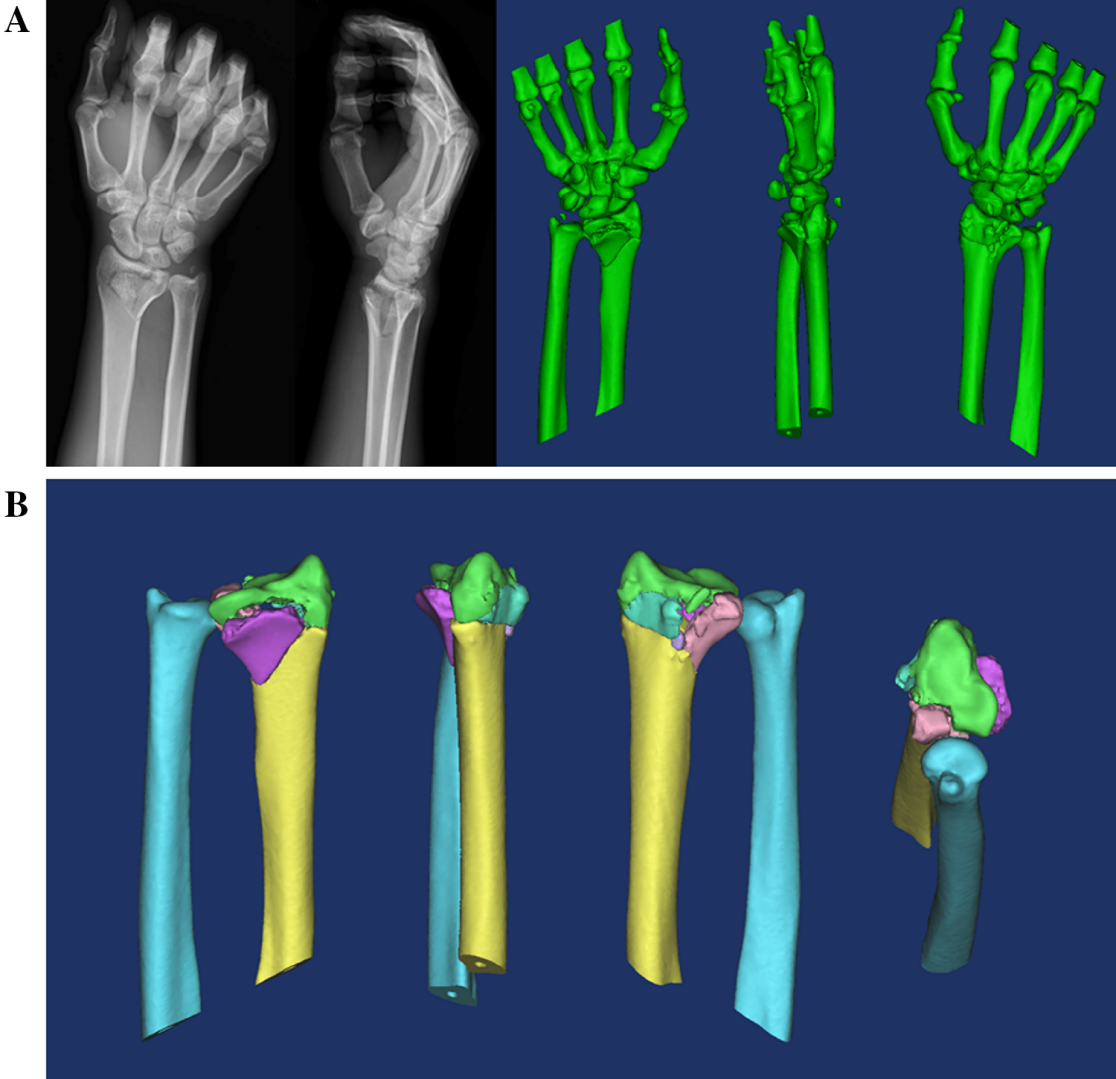
**a** Digital radiograph and 3D reconstruction of fracture. **b** Decomposition of fracture fragments, blue ulna, yellow shaft of radius, green upper fracture fragment of the radius, purple palmar fracture fragment of the radius, pink fracture fragment of the ulnar notch on the lower extremity of radius, blue in the radius dorsal fracture fragment of the radius

**Fig. 2:**
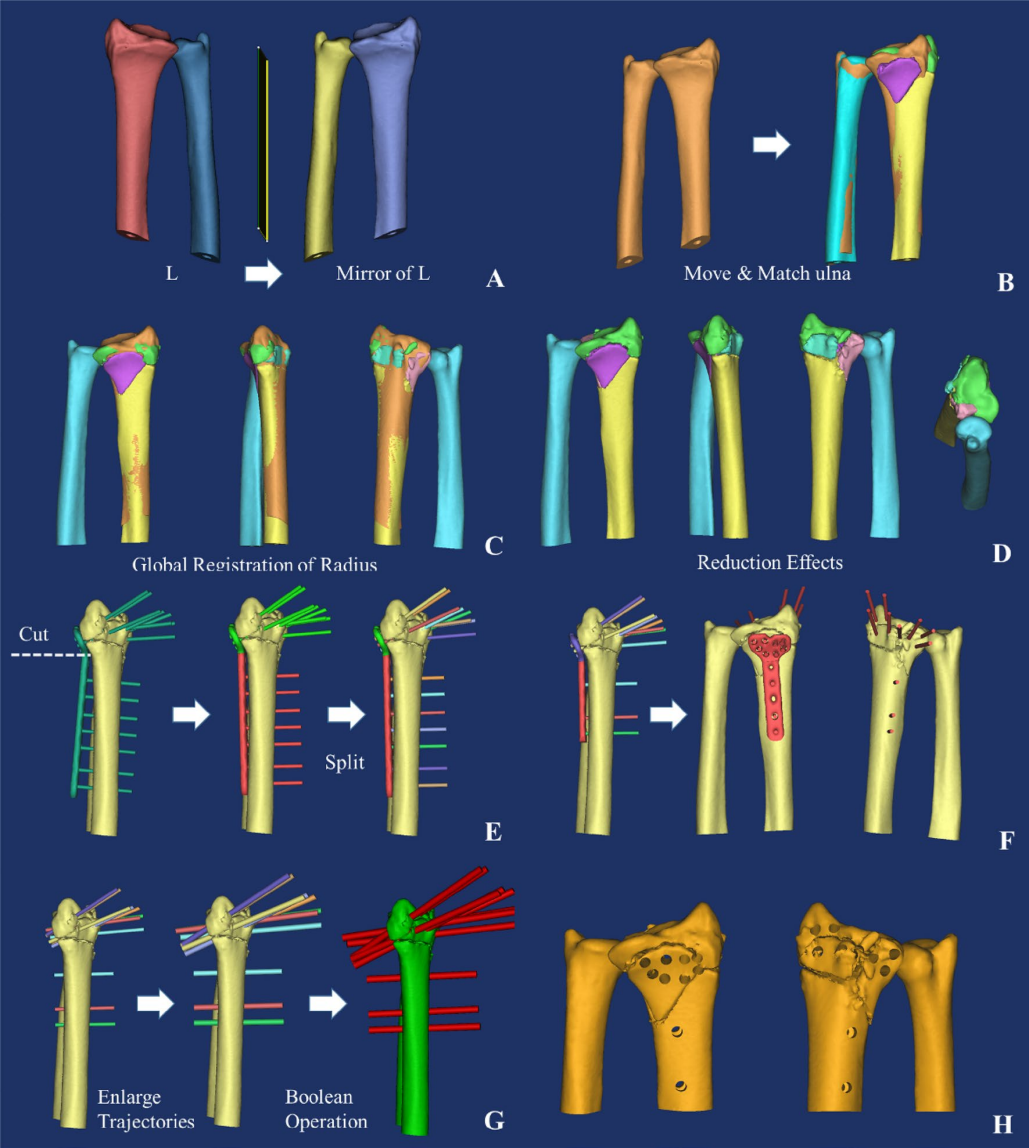
Design of the surgery. **a** Contralateral ulna mirror image of the healthy side (L), red radius of the healthy side, dark blue ulna of the healthy side, indigo radius of the mirror model, yellow ulna of the mirror model, **b** ulnar registration, orange mirror model, **c** radius registration, orange mirror model, **d** reset effect, blue ulna, yellow shaft of radius, green upper fracture fragment of the radius, purple palmar fracture fragment of the radius, pink fracture fragment of the ulnar notch on the lower extremity of radius, blue in the radius dorsal fracture fragment of the radius, **e** virtual pre-bending, **f** internal fixation scheme, red the virtual plate and screws, **g** radius model with screws, red the virtual screws, and **h** radius model with screw channels

(1) 3D reconstruction of digital radiograph and fracture was recreated virtually, and then the 3D fracture model was decomposed into independent fracture fragments by “Edit Mask in 3D”, as shown in Fig. 1a, b. (2) The mirror image of the ulna and radius of contralateral side was recreated virtually, as shown in Fig. 2a (3) ulna registration was operated with the function of “Move & Rotate” to register the contralateral ulna with the ipsilateral ulna, as shown in Fig. 2b (4) registration of the radius was performed through the function of “Align\Global Registration” registered the fractured blocks of the ipsilateral radius with the mirror radius one by one, as shown in Fig. 2c, and (5) manually fine-tuned according to the curvature of the fracture line and the bone transition of the fracture fragments, as shown in Fig. 2d.

#### Virtual internal fixation with a standard locking plate

(1) Operated with the function of “Move & Rotate” so that the nail of the locking plate was fixed to the fracture blocks. (2) Operated with the function of “Cut Orthogonal to Screen” to cut at the junction of head and the trunk of the locking plate, and then “Rotate” the trunk of the locking plate to fit the bone surface, as shown in Fig. 2e. (3) “Split” the 5-hole locking plate, i.e., decomposed the plate and the nail paths and partially removed nail paths according to the need of internal fixation, as shown in Fig. 2f. (4) Used the “Rescale Object” function to enlarge the diameter of the nail path to 3.8 mm, and (5) boolean operation was performed to obtain a radius model with screw channels, which would be used for accurate pre-bending of the locking plate, as shown in Fig. 2g, h.

#### Virtual radiograph

**Fig. 3:**
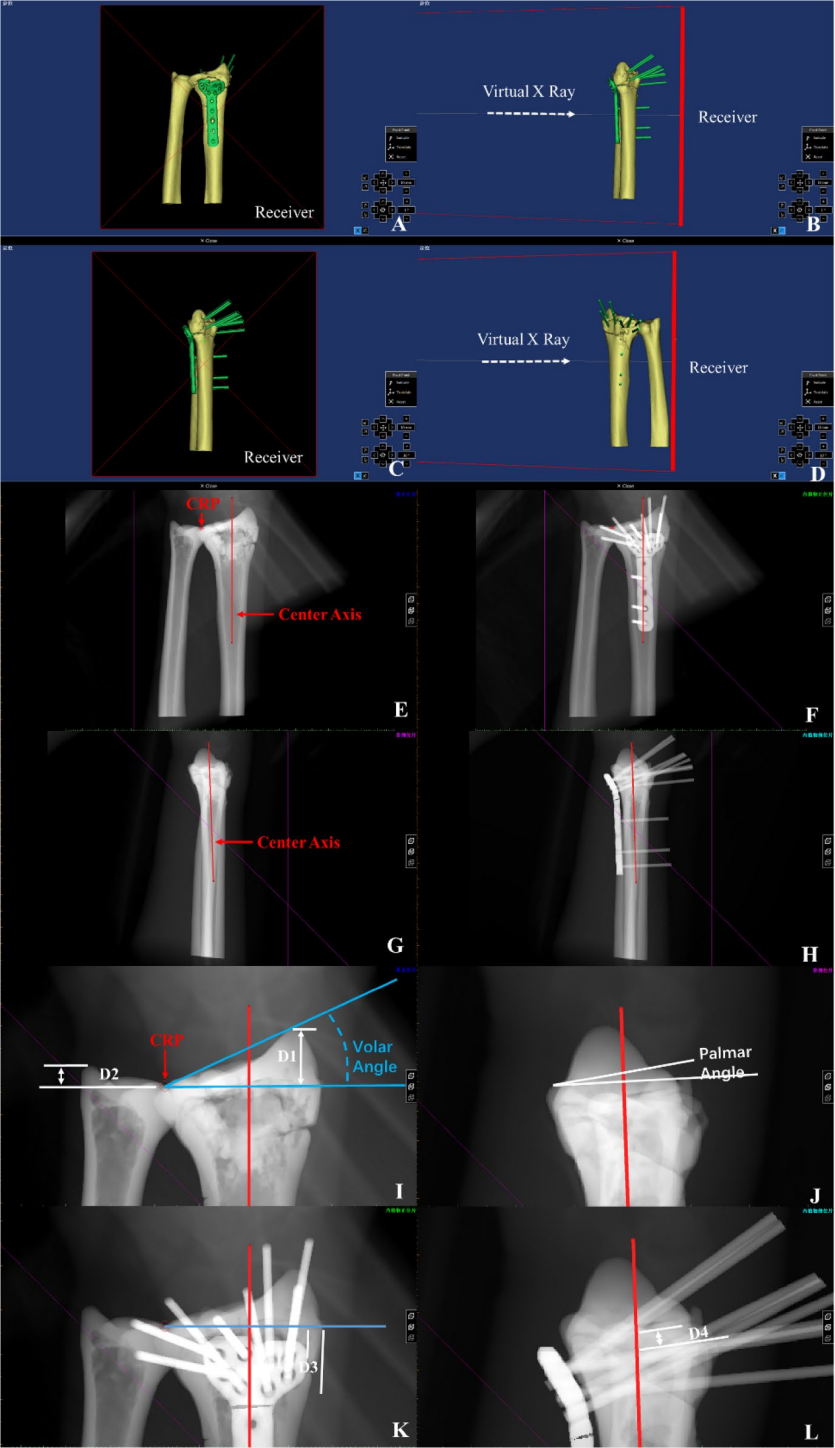
**a** and **b** The typical frontal imaging process, **c** and **d** the typical lateral imaging process, **e** and **f** the typical frontal images, **g** and **h** the typical lateral images, and **i**–**l** virtual X-ray measurement of different parameters

(1) Set the “Distance Source” to 3000 mm and the sampling board to 1000 * 1000 pixels (2) manual registration. Selected “X-Ray Object Registration”, and then checked “Initial Alignment” (3) adjusted the position of the positive film. The X-ray generator was projected from the palm side of the radius to the dorsal side. The long axis of the radius was in parallel with the sampling plate, as shown in Fig. 3a, b. (4) The position of the lateral imaging was adjusted by the same method, as shown in Fig. 3c, d, and (5) created virtual X-ray. Set the “Attenuation Coefficient, Image Data” to 0.15, the reset model to 0.35, and the locking plate at the highest 2.0, and generated a virtual positive side slice, as shown in Fig. 3e–h.

#### Virtual X-ray measurement

(1) Constructed the center reference point (CRP) with “Analyze\Create Point”, and built the central axis [23] with “CreateLine” (2) volar tilt (also called radial inclination), is an angle between two lines [24, 25], as shown in Fig. 3i (3) the radial length (or radial height) [26] is the distance between two lines, as D1 shown in Fig. 3i (4) ulnar variation [27] is the distance between two lines, as shown D2 in Fig. 3i (5) palmar tilt [26] is the angle between two lines, as shown in Fig. 3j (6) the positional parameter of D3 is shown in Fig. 3k, and (7) the distance between the key nail canal and joint surface D4 is shown in Fig. 3l.

#### The concept of U-arm assisted adjustment

**Fig. 4:**
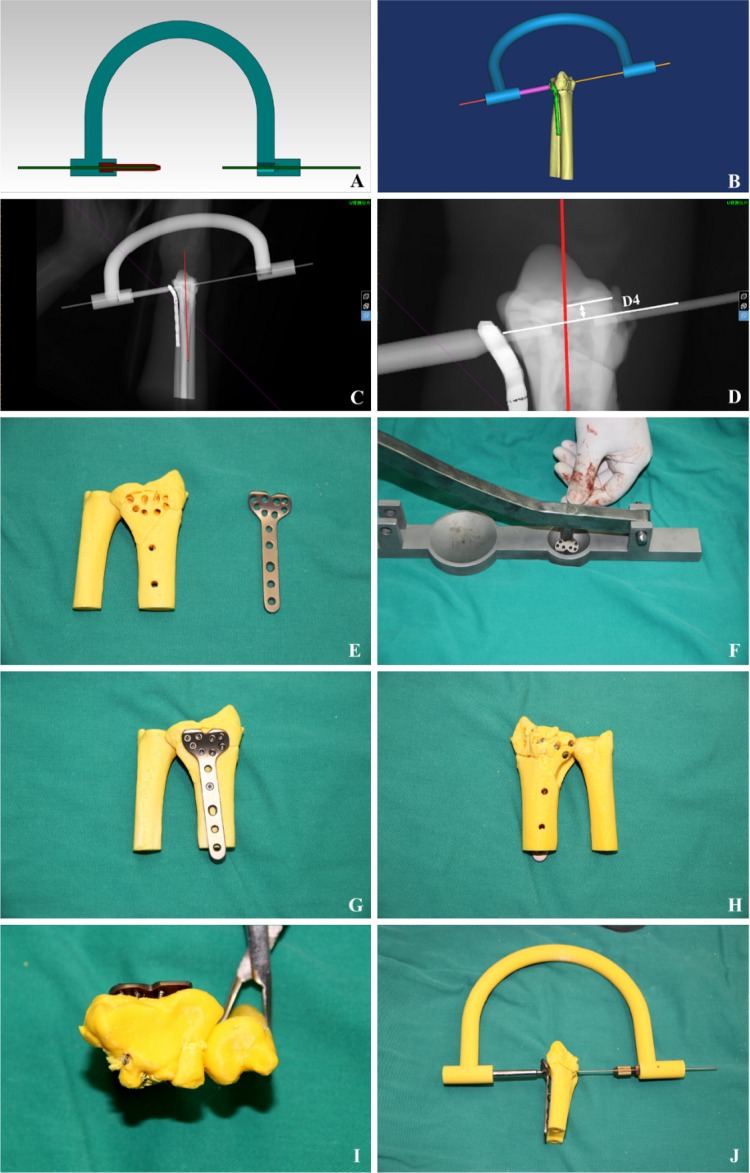
**a** schematic view of U-arm with locking guide and Kirschner wire, **b** virtual presentation of U-arm with locking guide (magenta), locking plate (green) and Kirschner wire, **c** typical X-ray image of U-arm with locking guide, locking plate and Kirschner wire, **d** schematic view of parameter D4, i.e., the key nail to joint surface distance, and **e**–**j** pre-bending of the locking plate

A U-arm was designed to form an integrated structure with the locking guide and the locking plate in this study, as shown in Fig. 4a. The U-arm could adjust the position of the locking plate in the radial palm surface (Fig. 4b, c) and adjust the distance between the “key nail” and the articular surface, as shown in Fig. 4d.

#### Accurately pre-bending the locking plate in reality

The real 3D printed radius model with screw channel could assist the accurately pre-bending locking plate, as shown in Fig. 4e–j. The criterion for precise pre-bending of the locking plate is that all locking screws can go through the screw channel when they are screwed into the locking hole and locked the plate tightly.

#### Surgical operation

**Fig. 5:**
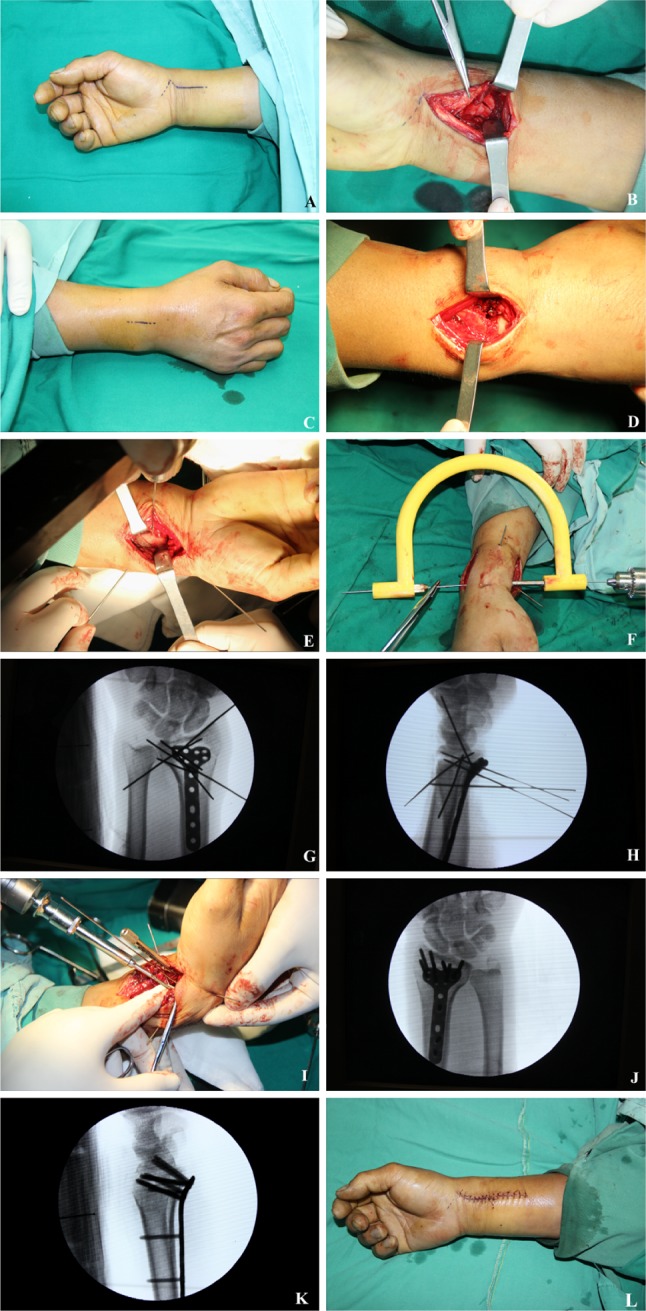
**a** and **b** The typical Henry approach, **c** and **d** the typical dorsal of the wrist approach, **e**–**h** U-arm assisted precise placement of key Kirschner wire, **i** image shows the process of screwing in the locking screw, **j** and **k** typical images caught by C arm that could help to re-check the fixation and measure the parameters, and **l** closed wound

Two surgical procedures were used (1) *Henry approach* cut the skin in front of the distal radius, cut the subcutaneous and deep fascia, separated brachioradialis tendon and flexor carpi radialis tendon, pulled the musculus flexor digitorum superficialis till saw the musculi pronator quadratus, partially cut the musculi pronator quadratus to expose the distal radius; performed manual reduction or reset with bone pry, as shown in Fig. 5a, b (2) *the dorsal of the wrist approach* opened the skin from the dorsal of the wrist, cut the subcutaneous and extensor retinaculum, revealed the tendon of extensor digitorum and the sheath of extensor indicis proprius tendon, separated extensor tendon, revealed and cut dorsal ligament of radiocarpal joint, revealed the dorsal part of the distal radius, as shown in Fig. 5c, d.

#### U-arm assisted precise internal fixation with locking plate

**Fig. 6:**
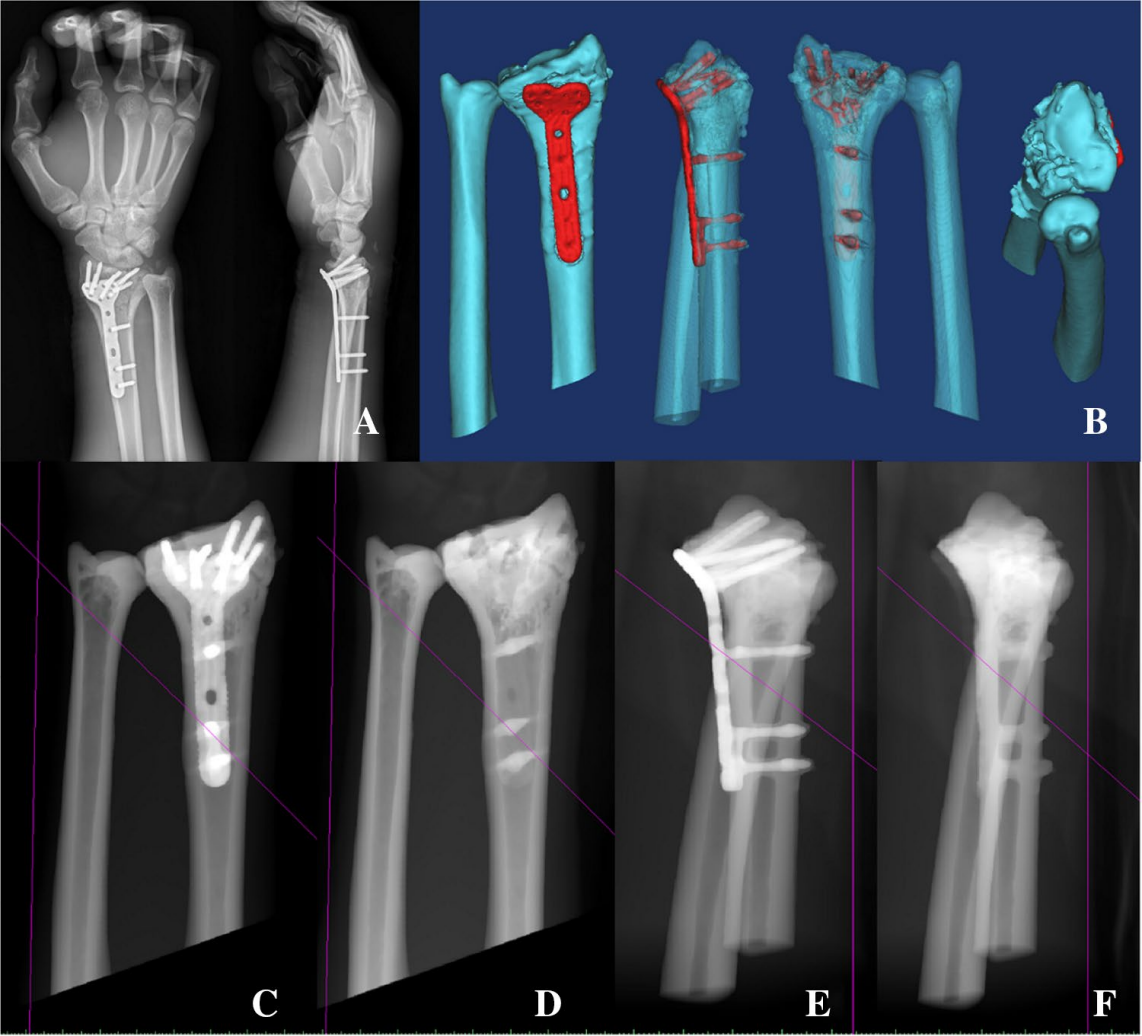
Typical real X-ray images (**a**, **c**–**f**) and virtual 3D reconstruction (**b**) demonstrated the final surgical effect (pink lines in **c**–**f** are system generated reference lines without any clinical implication)

(1) Reset with bone pry and then temporally fixed styloid process of radius and sigmoid cavity of radius with K-wires, as shown in Fig. 5e. (2) Inserted the locking plate, screwed into the locking guide, inserted the U arm, and inserted the K-wire to form the U-arm integrated structure. The position of the locking plate was adjusted by the U arm so that it was well fit to the bone surface, as shown in Fig. 5f, i (3) the frontal lateral C arm was used to check the internal fixation and reset. Figure 5 g, h showed that the locking plate and the key K-wires were in good position. Complete the internal fixation. Drilled holes, measured the depth and screw in the locking screw. Re-checked the internal fixation effect with the C-arm (i.e., measured the RSG parameters, including the volar tilt, palmar tilt, D1–4). The virtual X-ray measurement method was the same as DDG, as shown in Fig. 6j, k. Closed the wound, as shown in Fig. 5l. The typical internal fixation effect was shown in Fig. 6.

### Statistical analysis

Software SPSS13.0 was used in statistical analysis. The statistical differences of different parameters, including the volar tilt, palmar tilt, D1–4, of RSG and DDG were analyzed with t-test, p > 0.05 was considered as no statistical difference.

## Results

Twenty-one IDRF patients received the anatomical reduction and precise internal fixation through the approach of virtual X-ray and 3D printing. Since this approach was executed without apparent incidence of serious surgical complications, such as inaccurate reduction, accidental pierced-through of the screw to the wrist joint surface, functional deficiency or deformity, we concluded that the overall clinical outcome of this surgical approach was successful.

**Fig. 7:**
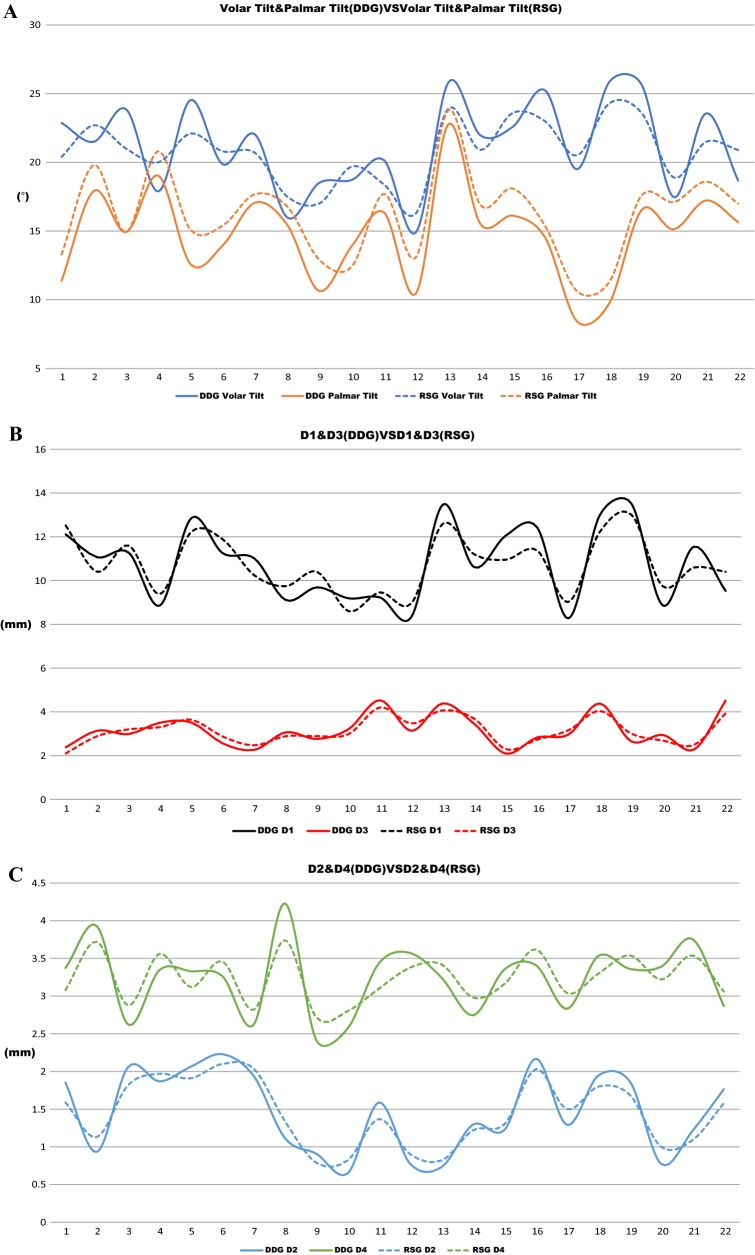
Presentation of individual value of each parameter. **a** Volar tilt and palmar tilt, **b** D1 and D3 and **c** D2 and D4, from each patient, both in the context of DDG and RSG

Consistent with this idea, all measured parameters, including the volar tilt, palmar tilt, D1–4, in RSG were within the expectation of virtual reduction (Fig. 7). It is worthy to stress that each parameter has been measured at least twice from each patient, i.e., one measurement was taken in the context of virtual reduction (DDG), and at least one measurement was taken in the context of real surgery (RSG). Comparing each parameter in two different contexts would help to quantitatively evaluate whether or not the real surgery is as successful as in the context of virtual reduction (the expectation).

Figure 7 depicted the individual value of each parameter, Fig. 7a included volar tilt and palmar tilt, Fig. 7b included D1 and D3 and Fig. 7c included D2 and D4 from each patient in the context of both DDG and RSG. The X-axis is the case sequence, and the Y-axis is the measured value, the solid line represents the DDG group, and the dotted line represents the RSG group. The solid lines and dotted lines are closely associated with each other in the context of each parameter, suggesting that the data collected form real surgery are very similar to that of the virtual reduction.

These data were further analyzed through statistic software (t-tests), and the final results were summarized in Table  2. Unsurprisingly, none of the measured parameters in RSG and DDG were significantly different, suggesting that real surgery was generally as successful as the virtual expectation (Table 2).

**Table 2:** Statistical analysis of the data from RSG and DDG

	DDG	RSG	t	P
D1 (mm)	10.78 ± 1.7	10.75 ± 1.30	0.07	0.47
D2 (mm)	1.46 ± 0.53	1.45 ± 0.44	0.14	0.44
D3 (mm)	3.16 ± 0.74	3.14 ± 0.59	0.11	0.45
D4 (mm)	3.23 ± 0.46	3.24 ± 0.31	− 0.02	0.49
Volar tilt (°)	21.24 ± 3.28	20.80 ± 2.24	0.51	0.31
Palmar tilt (°)	14.79 ± 3.32	16.19 ± 3.18	− 1.43	0.07

## Discussion

Anatomical reduction and precise fixation are still the most important concerns of orthopedic surgeons on IDRF surgery [6], which should include at least three aspects: (1) accurate evaluation (2) anatomical reduction, and (3) internal fixation to maintain the anatomical reduction. However, the final effect of the anatomical reduction has long been limited by the experience of the surgeon. Without reliable quantitative evaluation, or standardization, this situation would not be changed easily.

This study tested a digital surgery paradigm, which aimed to achieve the following objectives simultaneously: (1) virtual X-ray measurement as a way of quantitative evaluation of the anatomical reduction throughout the preoperative design/planning, intraoperative operation and postoperative analysis (2) use the small volar locking plate to achieve IDRF precise internal fixation. The anatomy of the distal radius is indeed very complex [1, 28]. For example, the carpal articular surface of distal radius interacts with navicular bone and lunate bone, which forms two depressions, i.e., scaphoid fossa and lunate fossa, over the cartilage surface. Ulnar side of distal radius participates in TFCC through articular disc, while the distal joint of the radius and ulna is also related to the rotation function of the forearm. In addition, the palm surface is adjacent to the carpal tunnel, which contains important structures, including the tendon of deep flexor of fingers, tendons of musculus flexor digitorum superficialis, flexor hallucis longus muscle and median nerve. Important structures such as long flexor and median nerve, and the posterior side of the distal radius are adjacent to the extensor tendon. Due to these complex structures, the anatomical reduction of IDRF becomes an extremely challenging issue.

The current criteria used to evaluate the final anatomical reduction of IDRF include: (1) the articular surface is completely rectified, the displacement is ≤ 2 mm (2) the height of the radius (D1) is restored. Dixon et al. [29] showed that the abnormal healing rate was 65% when the D1 reduction ≥ 3 mm, and could reach 73% if the D1 reduction ≥ 5 mm, which might shift the center of stress of the forearm to the ulna, causing distal joint movement disorders and even TFCC destruction [30] (3) Dario et al. [31] showed that ulnar variation and palmar tilt are the two most important anatomical reduction parameters, since these two indicators are closely related to the final surgical results, and palmar tilt is especially a very important and straightforward indicator for the reduction of distal radius fractures [24]. In this regards, Omokawa et al. [32] believed that arthroscopic surgery can better restore intra-articular fractures, find fracture fragments that are missed on preoperative X-ray and CT, detect and repair soft tissue damage, and even help to reasonably layout of steel plate screws. Similarly, Del Pinal [28] recommended the method of arthroscopic reconstruction of the distal radius fracture with the volar locking plate, but they considered that arthroscopy is only a supplement to the conventional method, only the classic technique is crucial for the treatment of distal radius fracture.

Regarding the precise internal fixation, currently, the locking plate is often used, and it is now commonly accepted that internal fixation with the volar locking plate is superior to non-surgical treatment, or percutaneous Kirschner wire fixation [33], or external fixation [34]. Mellstrand Navarro et al. [35] considered that the volar locking plate combined with Kirschner wire is a reasonable treatment for radial displacement fractures after low-energy trauma.

Regarding the locking plate, at present, the “small” volar locking plate is often used, which has the advantages of good internal fixation and less complications [36]. Simic et al. reported that the wrist and hand function recovered well using the dorsal *small* locking plate fixation. However, one problem of *small* locking plate design is that it often fails to achieve effective fixation of fractures distal to the “watershed” of the distal radius [37, 38]. Arora et al. [37] first reported the concept of “watershed” and they found that, when the implant is placed distal to the watershed, it is irritating or even causes fracture of the flexor tendon. Omokawa et al. [32] further elaborated the concept of watershed line, suggesting that it is an imaginary line between the two bone lines along the palm side of distal radius. In addition, within this area, when placing the volar locking plate, the protrusion of the distal radius is an important structure that we also need to pay attention to, so that we can avoid flexor tendon injury. Furthermore, Vaiss et al. [39] reported that when the volar locking plate was used, it was hard to locate the screw through the articular surface under conventional lateral front imaging. Some IDRF cases require the locking plate to be placed at a more distal position of distal radius to achieve the purpose of fixing the fractural blocks, but the *small* locking plate cannot easily cross the watershed. This is actually one of the problems we had to deal with in our study.

Having mentioned many potential problems, in this study, it was encouraging to find that no accidental pierced-through of the screw to the wrist joint surface, and more importantly, all the tested parameters in DDG and the RSG groups were not significant different; however, the palmar tilt in two groups was almost significantly different (p is only 0.07 in this case), suggesting the anatomical reduction of the palmar tilt is probably not as good as other parameters. The possible reasons are: (1) the Henry approach is used in the operation, the posterior of the wrist is less assessable, therefore, the dorsal reduction was not complete, and (2) the lateral view could potentially be interfered by the ulna and the carpal bone. Based on our experiences, to better quantitative evaluation, 2D X-ray measurement seem to be more suitable for the current IDRF anatomical reduction evaluation. It is because of that, in the context of 2D X-ray, it is easier to establish a unified measurement standard than the 3D model. In addition, there was a huge body of literatures that contain the reference of 2D X-ray measurement parameters. Finally, small community hospitals normally only have intraoperative C-arm, but not intraoperative CT.

To promote this approach, we would like to share more important tips in preoperative, intraoperative and postoperative procedure. For example (1) preoperatively, after the severe DRF, the normal positional relationship between the ulnar and radial bones was often lost. In this case, virtual reduction is normally dependent on the mirror image of the unaffected side. (2) If there is no mirror model or bilateral DRFs happen, virtual reduction should be performed with the distal radius of the distal joint as the starting point (3) it is necessary to accurately pre-bend the locking plate in the 3D printed out model with screw channels, and test the locking screw into the plate to see if there is any obstruction, preoperatively. (4) Intraoperatively, it is necessary to rely on C-arm data to guide anatomical reduction. (5) U-arm can help to achieve a good overall internal fixation of the distal radius fracture while accurately implanting the key Kirschner wire. (6) The correct layout of the bone plate and the nail path could be achieved when both D3 and D4 parameters are correct. (7) During the operation, the Kirschner wire should be placed about 5 mm into the bone with the U-arm. (8) Drill holes only after the position of the locking plate and the Kirschner wire is completely correct, determined by the C-arm. This can provide the surgeon with multiple opportunities to repent, making the internal fixation program closer to preoperative planning. (9) In this study, the Henry approach and the dorsal approach of the wrist were used, the position of the key Kirschner wire was accurately determined under direct vision during the operation. (10) For quality control, we should reduce the virtual X-ray magnification, i.e., the radius should be as close as possible to the sampling plate, and the bulb (source of X-ray) should be kept away from the radius, so that the measured data is closer to the true value. (11) Even though the quantitative indicators and specific methods of anatomical reduction in this paper were based on the traditional classical method, the procedure was slightly improved according to the surgeon’s habits.

Perspectives on current challenges: (1) virtual fracture reduction is the rate-limiting step of this method, yet there is no available automatic fracture reduction software so far (2) C-arm cannot guarantee 100% clear observation and anatomical reduction; therefore, sometimes it is necessary to cut the joint capsule to directly observe and perform the reduction (3) during preoperative planning, if the selected standard locking plate cannot meet the requirements after the pre-bending, other locking plate should be considered, and (4) virtual X-ray technology can also be used for internal fixation of other bones.

## Conclusion

The virtual 3D printing and X-ray measurement of anatomical reduction parameters can provide quantitative references for anatomical reduction and accurate internal fixation of intra-articular fractures of the distal radius.
